# Fabric-like Electrospun PVAc–Graphene Nanofiber Webs as Wearable and Degradable Piezocapacitive Sensors

**DOI:** 10.1021/acsami.3c03113

**Published:** 2023-04-25

**Authors:** Debarun Sengupta, Liqiang Lu, Diego Ribas Gomes, Bayu Jayawardhana, Yutao Pei, Ajay Giri Prakash Kottapalli

**Affiliations:** † Department of Advanced Production Engineering (APE), Engineering and Technology Institute Groningen (ENTEG), University of Groningen, Groningen 9747 AG, The Netherlands; ‡ Department of Discrete Technology and Production Automation, Engineering and Technology Institute Groningen, Faculty of Science and Engineering, University of Groningen, Groningen 9747 AG, The Netherlands

**Keywords:** nanofibers, piezocapacitive, graphene, wearables, degradable sensors, flexible electronics

## Abstract

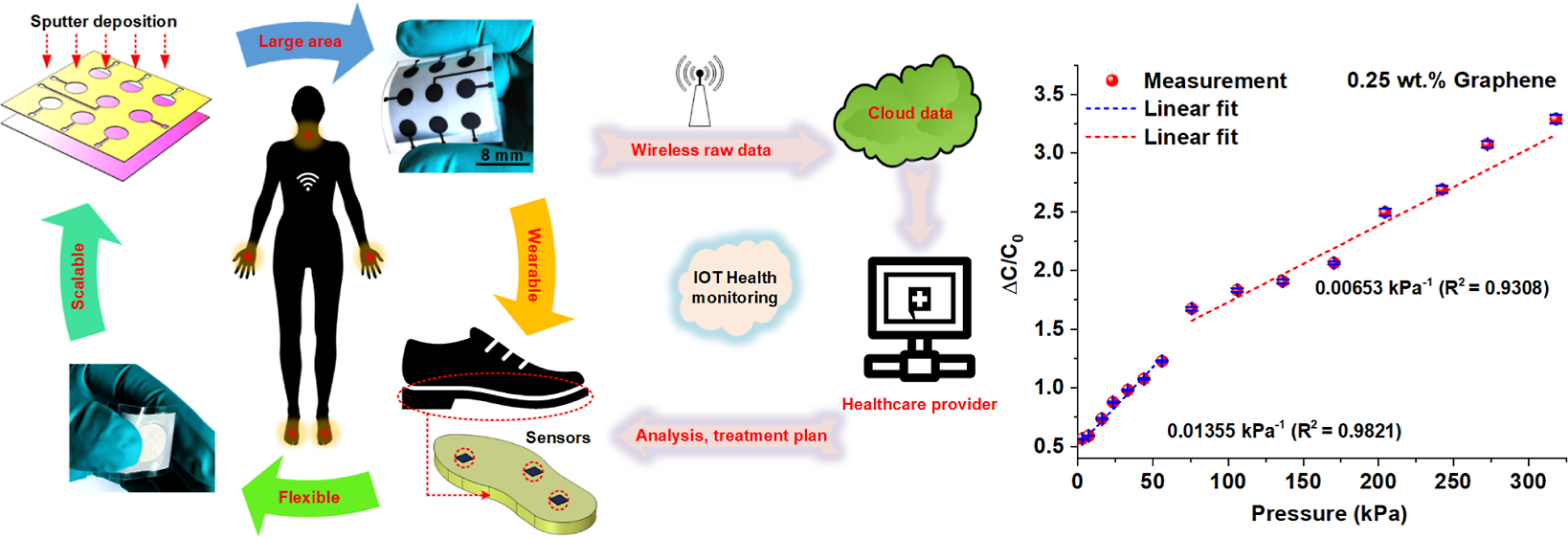

Flexible piezocapacitive sensors utilizing nanomaterial–polymer composite-based nanofibrous membranes offer an attractive alternative to more traditional piezoelectric and piezoresistive wearable sensors owing to their ultralow powered nature, fast response, low hysteresis, and insensitivity to temperature change. In this work, we propose a facile method of fabricating electrospun graphene-dispersed PVAc nanofibrous membrane-based piezocapacitive sensors for applications in IoT-enabled wearables and human physiological function monitoring. A series of electrical and material characterization experiments were conducted on both the pristine and graphene-dispersed PVAc nanofibers to understand the effect of graphene addition on nanofiber morphology, dielectric response, and pressure sensing performance. Dynamic uniaxial pressure sensing performance evaluation tests were conducted on the pristine and graphene-loaded PVAc nanofibrous membrane-based sensors for understanding the effect of two-dimensional (2D) nanofiller addition on pressure sensing performance. A marked increase in the dielectric constant and pressure sensing performance was observed for graphene-loaded spin coated membrane and nanofiber webs respectively, and subsequently the micro dipole formation model was invoked to explain the nanofiller-induced dielectric constant enhancement. The robustness and reliability of the sensor have been underscored by conducting accelerated lifetime assessment experiments entailing at least 3000 cycles of periodic tactile force loading. A series of tests involving human physiological parameter monitoring were conducted to underscore the applicability of the proposed sensor for IoT-enabled personalized health care, soft robotics, and next-generation prosthetic devices. Finally, the easy degradability of the sensing elements is demonstrated to emphasize their suitability for transient electronics applications.

## Introduction

The last two decades have witnessed a significant growth in the wearable-focused consumer electronics industry, and the rise in the consumption of smart watches and accessories is a testament to that fact. Especially, with the rapid incremental progress being made every day, the field of wearable electronics is hovering around the brink of science fiction. The Internet of Things (IoT) revolution in the last decade has pushed wearable sensors to the forefront of research interest across the globe. Today, the demands for seamless integration of wearable sensors which can monitor vital physiological parameters have skyrocketed, and this dramatic rise in demand has been partially fueled by the onslaught of the COVID-19 pandemic since late 2019. The need of the hour is reliable, facile, low-powered, and inexpensive sensors fit for seamless integration into various wearable apparel that will enable nonintrusive acquisition of vital health parameters with the aim of improving the quality of life of the user. However, for any wearable sensor technology to be considered seriously in the healthcare domain, parameters such as sensitivity, ease of use, cost effectiveness, long term-reliability, and, most importantly, a low power budget, are of paramount importance. In addition to applications in human physiological function monitoring, flexible sensors are also of great importance in developing artificial skin for next-generation prostheses, soft human–machine interfaces, robotics-assisted medical facilities, and other similar areas.

Historically, strain and pressure sensing devices have relied on different sensing mechanisms, namely piezo-resistive,^1−7^ piezoelectric,^8−13^ and capacitive.^14−17^ Other mechanisms utilizing transistors for pressure sensing have also been reported in the literature.^18−20^ More recently, the triboelectric sensing mechanism has also been employed in the development of pressure and strain sensors.^21,22^ Though the piezoelectric sensing mechanism offers the advantage of self-powered sensing, their inability to sense static pressure places a major hindrance on their applications in tasks requiring both static and dynamic pressure sensing. In contrast, piezoresistive sensing offers functionality in a wide range of pressure sensing conditions, including static, quasi-static, and dynamic pressure/strain sensing. However, resistive sensors have the inherent problems of thermal drift, high hysteresis, and a relatively higher power budget. Finally, the fundamental problem with transistor-based sensors lies in their complex fabrication process and associated circuitry. Piezocapacitive sensing offers an attractive alternative to more traditional piezoelectric and piezoresistive sensing owing to its ultralow-powered nature due to the lack of flow of DC current. Furthermore, piezocapacitive sensors offer added advantages, such as superior stability, temperature independence, low hysteresis, and fast response time.

Traditional parallel-plate electrode-based capacitive pressure sensors comprise two conductive electrodes separated by a dielectric medium. Upon application of external pressure, the distance between the two electrodes changes, thereby leading to a change in capacitance that can be measured with an external circuit. The structural property of the dielectric layer between the two electrodes has a profound impact on the sensing performance of the sensor. Though traditional capacitive pressure sensors rely on the changing distance (*d*) between the electrodes, more recently, methods are being investigated to achieve pressure-induced dielectric constant (ε_*r*_) changes to achieve enhanced pressure sensitivity.^23,24^ Furthermore, it is desirable that the dielectric layer has a low compressive modulus, which will further contribute toward enhancing the pressure sensitivity. In the past, elastomeric materials with microstructured air voids have been employed for enhancing the sensing performance of capacitive sensors.^23,25^ For instance, Mannsfeld et al. demonstrated a microstructured polydimethylsiloxane (PDMS)-based large-area flexible capacitive sensor array with fast response.^23^ More recently, researchers have employed ionic hydrogels for developing capacitive sensors for pressure sensing, humidity sensing, and human physiological parameters monitoring.^26,27^ Ding et al. reported a polyvinyl alcohol-cellulose nanofiber-based biocompatible, self-healing, breathable, stretchable, and degradable organohydrogel ionic skin with superior humidity responsiveness.^26^

In the case of capacitive sensors employing dielectric layers with air voids, the initial effective dielectric constant is a combination of the dielectric constants of air and the porous/microstructured material. Upon application of an external pressure, the gap (*d*) between the electrodes reduces, squeezing the sandwiched dielectric layer and replacing the low permittivity air voids with high permittivity dielectric material in the process. The change in the dielectric constant with applied pressure contributes to the enhanced sensing performance. Though dielectric elastomeric structures with micropatterned air voids offer an attractive proposition, the inherent complexity of fabrication involving multistep patterning processes and reliance on expensive machinery place constraints on small and medium-sized enterprises to develop and commercialize them.

Electrospinning offers an attractive alternative to traditional microfabrication techniques for fabricating nanofibrous dielectric layers with air voids owing to the simplicity of the processing steps. The porous nature of the electrospun nanofibrous scaffolds renders them with a low compression modulus and high deformability, which is desired in wearable sensing applications. Electrospinning has evolved to be the preferred method for fabricating large-area, flexible, and porous membranes. Due to their inexpensive and facile nature, recently, there has been renewed interest in utilizing electrospun nanofibrous membranes for flexible capacitive pressure sensing.^24,28−30^ Zhu et al. reported a polyimide nanofibrous membrane-based flexible capacitive pressure sensor with a superior sensitivity of 2.202 kPa^–1^ in the low pressure range ∼3.5–4.1 Pa.^28^ However, issues such as the low dielectric constant of polymeric materials limit their usage in wearables requiring high sensitivity and a low detection threshold. Incorporation of nanomaterial fillers in polymeric materials has proven to improve their dielectric performance.^31−36^

Piezocapacitive sensors utilizing polymeric nanofibers with nanomaterial fillers are relatively new and must be explored further to assess their suitability for applications in wearables. Although polymeric nanofibers enable the facile fabrication of flexible and pliant structures with low compression modulus, their low dielectric permittivity limits their usage for applications in supercapacitors and piezocapacitive sensors. Incorporation of fillers in polymeric materials near the percolation threshold has proven to enhance the dielectric properties of the composites.^37^ Especially, the incorporation of nanomaterial fillers, such as carbon nanotubes (CNTs) and graphene and MXene nanoflakes, has proven to enhance the dielectric performance of the polymeric materials.^31−36^ Furthermore, the addition of nanomaterial fillers in low volume fractions has the potential to enhance the dielectric property of the polymeric materials without compromising their flexibility or mechanical properties. Yang et al. reported CNT-polyvinylidene fluoride (PVDF) nanofibrous membrane-based flexible piezocapacitive tactile sensors for wearable applications.^36^ However, due to the one-dimensional (1-D) nature of CNTs, they have a lesser surface area in comparison to 2D materials with a larger specific surface area and tend to form agglomerates that lead to poor dispersion in nanofibers and can subsequently reduce the mechanical performance of the membranes. Incorporation of 2-D materials like Graphene and MXene (Ti_3_C_2_T_*x*_) can potentially alleviate the problems associated with lower-dimension nanofillers and lead to the fabrication of better performing dielectric layers. Previously, Sharma et al. reported that MXene dispersed polyvinylidene fluoride-trifluoroethylene (PVDF-TrFe) nanofibers for developing high performance flexible and wearable capacitive sensors.^33^ The harsh acid etching used for synthesizing MXene sheets led to defect sites in the flakes that formed centers of oxidation, leading to the loss of conductivity.^38^ Furthermore, the presence of hydroxyl groups on the MXene surface (to achieve better dispersion in water) resulted in a reaction with dissolved oxygen and water molecules, thereby causing oxidation and a subsequent loss of conductive properties.^38^ In contrast, graphene can prove to be an attractive alternative owing to its superior stability coupled with excellent electrical and mechanical properties.

In contrast to the previous works utilizing materials like PVDF and PVDF-TrFe, in this research, we have employed PVAc, an inexpensive and degradable polymeric material (also referred to as white glue), for synthesizing the nanofibrous membranes. PVAc is a rubbery, thermoplastic synthetic nontoxic^39,40^ polymer having the formula (C_4_H_6_O_2_)_*n*_, which makes it a promising candidate for usage in sensors targeted for human physiological monitoring applications.

**Figure 1:**
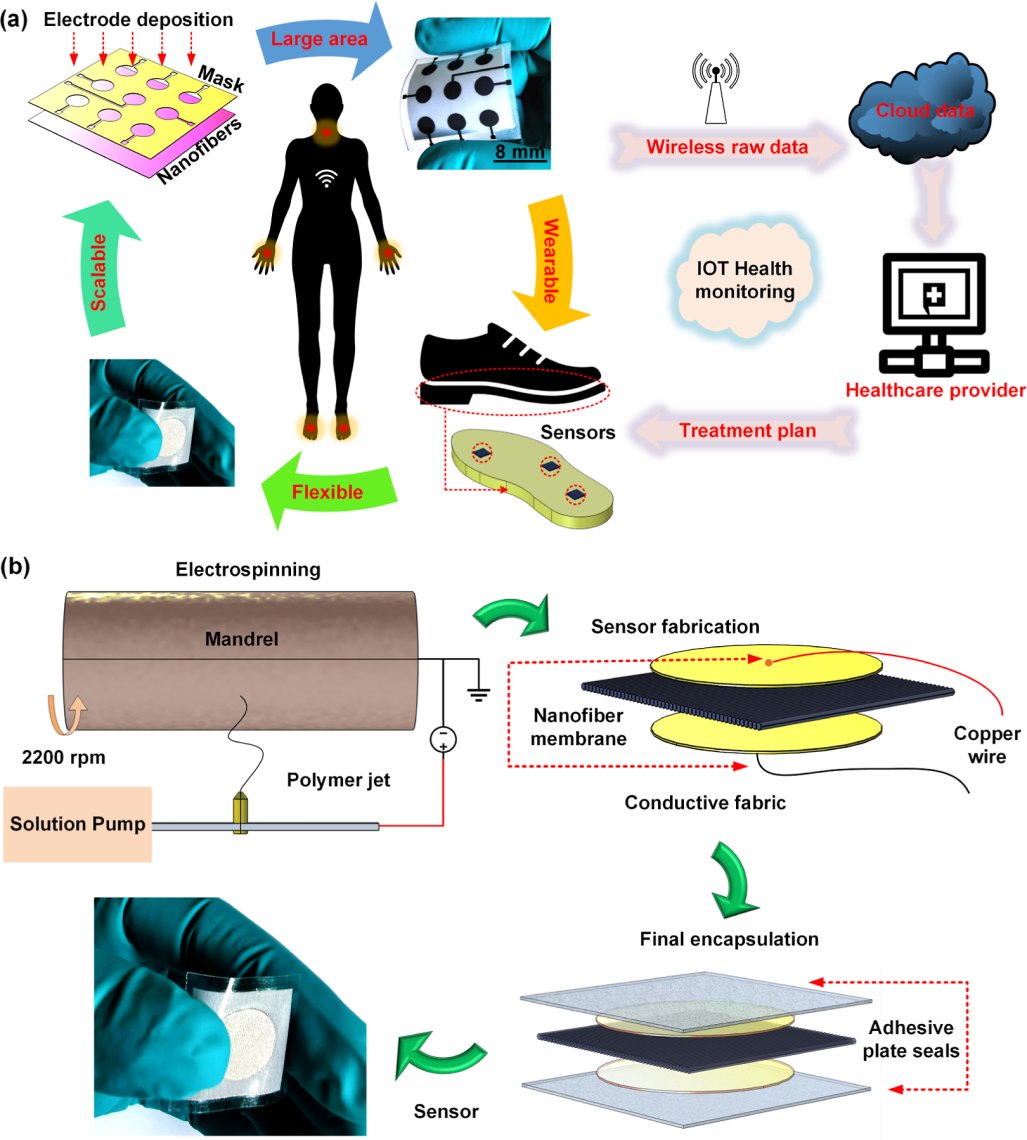
Electrospinning and sensor fabrication flow: (a) application of graphene–PVAc nanofiber sensors for next-generation wearable-enabled telemedicine and rehabilitation; (b) schematic representation of the process flow involved in the fabrication of fabric-like PVAc–graphene nanofiber sensors.

In this work, we propose a facile method for fabricating graphene-dispersed polyvinyl acetate (PVAc) electrospun nanofiber-based piezocapacitive sensors for flexible and wearable electronics applications. A method entailing the sandwiching of graphene-dispersed PVAc nanofibrous membranes between two layers of flexible fabric electrodes for fabricating a fully flexible and apparel-integrable sensor is demonstrated. Dielectric response assessment tests are conducted on both the pristine PVAc and graphene-dispersed PVAc membranes (fabricated by spin coating) to understand the effect of the addition of graphene nanofillers on the dielectric constant enhancement. The microcapacitor network theory and microscopic dipole formation model (interfacial polarization) have been invoked to explain the dielectric response enhancement. Material and morphological characterization experiments are conducted on both the pristine and graphene-loaded PVAc nanofibers to understand the effect of graphene dispersion on nanofiber properties and morphologies. Electromechanical characterization experiments are conducted on the pristine and graphene-loaded PVAc nanofibrous membrane-based piezocapacitive sensors for assessing the effect of graphene nanofiller addition on pressure sensing performance. The 0.25 wt % graphene-loaded PVAc nanofibrous membrane-based piezocapacitive sensor demonstrated superior performance with two distinct linear regimes and pressure sensitivity figures of 0.01355 kPa^−1^(∼2.73–56.06 kPa) and 0.00653 kPa^−1^(∼75.76–318.18 kPa). To stress the robustness and reliability of the sensors for real-life applications, a series of accelerated lifetime assessment tests involving at least 3000 cycles of tactile force loading (2.7, 10, and 20 N) are demonstrated. The 0.25 wt % graphene-dispersed PVAc nanofibrous membrane-based piezocapacitive sensors are chosen for the subsequent applications demonstrated in this work. The suitability of the sensors for applications in wearables and human physiological monitoring has been validated by conducting a series of demonstration experiments involving muscle movement detection, respiratory rate monitoring, sensorized smart shoe insoles for gait detection, and a posture sensing smart chair for encouraging pressure-relieving maneuvers. The readiness of the proposed piezocapacitive sensors for IoT integration has been underscored through custom-written embedded software programs (implemented on Arduino Uno and Nano platforms) and subsequent applications in wireless gait monitoring smart insoles and posture sensing smart chairs. Finally, the degradability and subsequent recyclability of the sensor have been demonstrated to emphasize the relevance of the sensor for the circular electronics initiative theme. The facile sensor reported in this work would inspire a future telemedicine ecosystem comprising of low-powered, highly sensitive piezocapacitive sensor networks coupled with IoT hardware for continuous monitoring of human physiological vitals, thereby reducing the load on existing healthcare infrastructure, as conceptually shown by the schematic in Figure 1a.

## Results

### Fabrication and Material Characterization of Graphene–PVAc Nanofibers

#### Synthesis of Nanofibrous Membranes and Sensor Fabrication

In this work, electrospinning was employed for fabricating pristine PVAc nanofibers. To understand the effect of nanomaterial fillers on the dielectric constant/permittivity, commercially acquired graphene nanoflake dispersion was added to the pristine PVAc polymer solution to achieve 0.1, 0.25, and 0.50% weight of graphene nanofillers in PVAc. Electrospinning was subsequently conducted to synthesize the graphene-loaded PVAc nanofibers. More details of the polymer solution preparation and subsequent electrospinning process are furnished in the Experimental Section.

The as-spun nanofibers on aluminum foil substrate are transferred from the rotating mandrel collector and diced into 2 cm × 2 cm sections. To develop all flexible, wearable, and apparel-integrable sensors, the diced nanofibrous membranes were sandwiched between two layers of circular silver-plated nylon fabric bonded to single-strand copper wires for subsequent electrical connections. A complete encapsulation was achieved by employing two layers of optically clear adhesive plate seals. Figure 1b schematically represents the process steps involved in the electrospinning of the pristine PVAc and graphene-loaded PVAc nanofibrous membranes. Further details regarding the flexible sensor fabrication are furnished in the Experimental Section.

#### Morphological and Material Characterization

**Figure 2:**
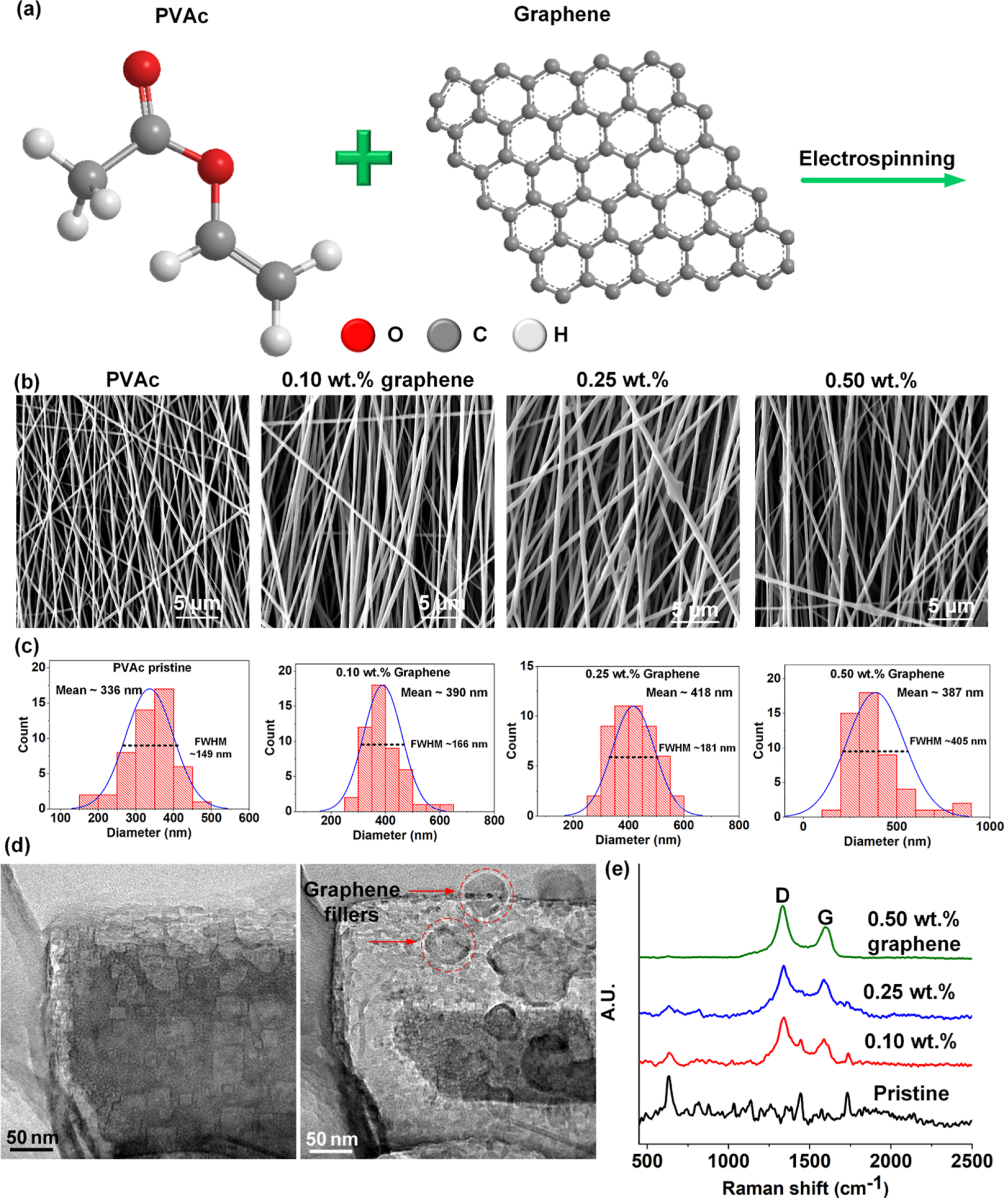
Material characterization: (a) showing the chemical structure of PVAc and graphene; (b) SEM micrographs comparing the morphologies of the pristine PVAc nanofibers with the graphene-loaded PVAc nanofibers; (c) histograms comparing diameter distributions of the pristine PVAc nanofibers with graphene–PVAc nanofibers; (d) TEM micrographs showing the dispersion of graphene nanoflakes in a PVAc nanofiber; (e) Raman spectra comparing the pristine PVAc nanofiber bundle with graphene–PVAc nanofiber bundles.

In order to understand the effect of the addition of graphene nanofillers on the dielectric characteristics of the polymer composite, pristine PVAc and graphene-loaded PVAc nanofibers were synthesized by electrospinning. Graphene was added in varying concentrations (0.10, 0.25, and 0.50 wt %) to a PVAc polymer solution, and electrospinning was conducted to achieve graphene-loaded PVAc nanofibers. Figure 2a shows the molecular structures of PVAc and Graphene. To understand the effect of graphene fillers on the morphological properties of the nanofibers, field emission scanning electron microscope (FESEM) images were acquired. The micrographs in Figure 2b compare the morphology of the pristine PVAc nanofibers with the graphene-loaded samples. For higher loadings, nanofiller agglomerates were observed, as clearly seen in the micrograph (0.50 wt %) in Figure 2b. The diameter distributions of the nanofibers are compared, as shown by the plots in Figure 2c. The mean diameters of the nanofibers were determined as 336.12 (pristine PVAc), 389.74 (0.10 wt % graphene), 417.64 (0.25 wt % graphene), and 386.62 nm (0.50 wt % graphene). The average diameters of the nanofibers were observed to have increased with graphene loading till 0.25 wt %. Furthermore, the plot in Figure 2c compares the full width at half maximum (FWHM) for the nanofiber samples. An increase in the FWHM with increasing graphene loading was observed. The 0.50 wt % graphene-loaded PVAc nanofiber sample demonstrated the highest FWHM of 405 nm signifying poor uniformity in comparison to the other samples (Figure 2c). The observed reduction in the mean diameter of the 0.50 wt % graphene-loaded PVAc can possibly be attributed to the nonuniform diameter distribution, where nanofibers with graphene agglomerates have a large diameter in comparison to thinner nanofibers without agglomerates in the sample. Transmission electron microscopy (TEM) imaging was conducted to observe the dispersion of graphene nanofillers inside PVAc nanofibers. Progressive irradiation resulted in damage to the PVAc matrix, thus exposing the graphene nanofillers and causing them to dynamically rearrange (frames can be seen in Video SV1). The micrographs in Figure 2d show the dispersion of graphene nanofillers inside an isolated PVAc nanofiber section. The micrograph on the left (Figure 2d) shows graphene fillers embedded inside the isolated PVAc nanofiber fragment. The micrograph on the right (Figure 2d) shows the exposed graphene nanofillers after the nanofiber fragment is damaged with the electron beam, subsequently releasing them. Raman analysis was performed on the pristine and the graphene-loaded nanofibers to confirm the dispersions and the structures of the graphene nanofillers. As shown in Figure 2e, in the case of the pristine PVAc nanofibers, the normalized Raman spectrum does not demonstrate any noticeable bands from graphene; namely, all the bands are from PVAc. In comparison, the spectra for all graphene-loaded samples demonstrated a prominent D band (at 1340 cm^–1^) that is triggered by the defects and a G band (at 1596 cm^–1^) for the characteristic of graphite/graphene. The intensity ratio of the D band to the G band is 1.10, indicating that graphene contains many defects.

Lattice defects are inevitably introduced during the production/synthesis process of graphene, which lead to deviations from its theoretical mechanical and electrical properties. The defects introduced during the synthesis process lead to changes in the bond length of the interatomic valence bonds and potentially to changes in their conductivity.^41^ However, the graphene fillers used in this work are intended to enhance the dielectric constant of the composites by harnessing the phenomenon of interfacial polarization. As such, eventual changes in the conductivity of the graphene nanofillers as compared to the defect-free case do not significantly impact the pressure sensing performance of the resulting sensors. Furthermore, the relative intensity of the peaks improved progressively with increasing the graphene loadings, as observed from the plots in Figure 2e. The Raman spectral analysis (qualitatively) confirms the presence of graphene nanofillers in the PVAc nanofibers.

#### Mechanism for Dielectric Constant Enhancement

**Figure 3:**
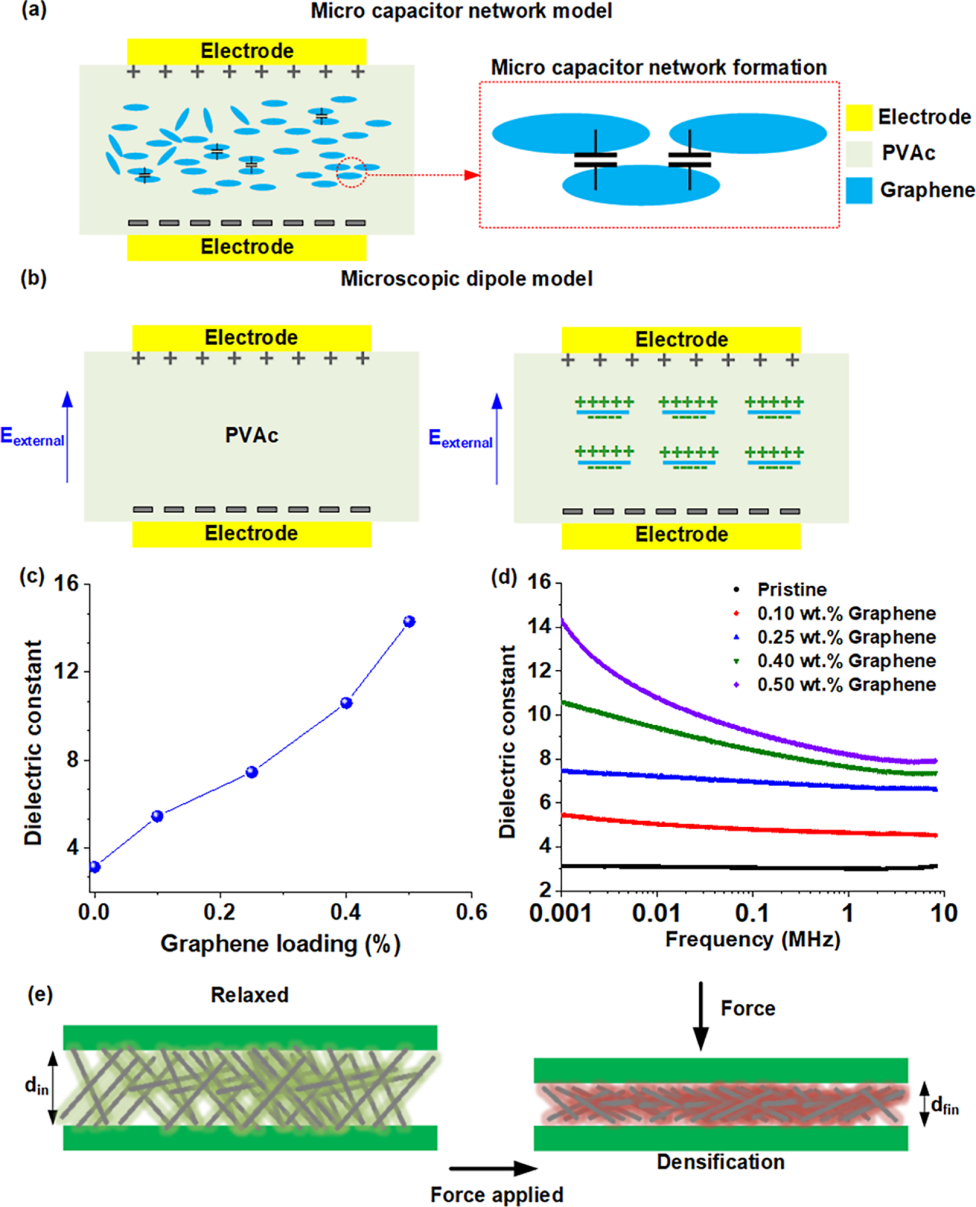
Dielectric response characterization: (a) schematic representation of the microcapacitor network model; (b) schematic representation of the microscopic dipole formation model; (c) plot showing the dielectric constant with respect to graphene loading; (d) plots comparing the dielectric frequency response of the spin-coated pristine PVAc membrane with the graphene–PVAc composite spin-coated membrane; (e) schematic representation of the working mechanism behind the piezocapacitive sensor.

Research works conducted in the past have established that the incorporation of nanomaterial fillers in polymeric materials generally improves their dielectric performance.^31−36^ From the perspective of percolation theory, in the case of conductive fillers dispersed in nonconductive polymers, the dielectric constant (κ) of nanomaterial-polymer composites follows the relation: κ  κ_p_*|f*–*f*_c_*|*^–*s*^, where κ_p_ represents the dielectric constant of the insulating polymer matrix, *s* is an exponent with a value of ∼1, *f* represents the fractional volume of the nanofiller in the polymer matrix, and *f*_c_ represents the critical fractional volume at percolation threshold.^42^ A number of theories have been hypothesized to explain the phenomenon of dielectric constant enhancement in the case of insulating polymeric materials loaded with nanomaterial fillers.^43−45^ In particular, the microcapacitor network formation theory has been invoked by several researchers to explain the giant enhancement of dielectric constant near percolation threshold.^33,42,43,46^ The sketch in Figure 3a schematically represents the microcapacitor network theory. When the volume fraction of nanofillers approaches the percolation threshold (below *f*_c_), several microcapacitors are formed by neighboring conductive nanofillers separated by very thin layers of the polymer insulator. Each of these individual microcapacitors demonstrate a very high capacitance figure and subsequently contribute to the overall enhancement of the dielectric constant of the composite.^42,43^ Another mechanism that also contributes to the dielectric constant enhancement is the microscopic dipole formation model.^34,47^ In this case, application of an external electric field to the nanomaterial polymer composite leads to charge accumulation at the interface between the nanomaterial fillers and the polymer matrix, which subsequently leads to interfacial polarization (Maxwell–Wagner-Sillars polarization) as depicted by the schematic in Figure 3b. To assess the effect of graphene nanofiller addition on the dielectric constant, composite membranes with 0.1, 0.25, 0.4, and 0.5 wt % graphene filler concentration in PVAc were fabricated by spin coating and subjected to experiments involving dielectric response vs frequency sweep (in the range ∼1 kHz–8 MHz).

The plot in Figure 3c shows the dielectric constant as a function of graphene loading at 1 kHz (1 V) excitation. Frequency sweeps (in the range ∼1 kHz–8 MHz) were conducted on the pristine and graphene-loaded PVAc films, as shown in the plot in Figure 3d. As observed from both the plots, the dielectric constant increases with increasing graphene loading. Given that the concentrations of the graphene nanofillers in the graphene-PVAc composite membranes are well below the percolation threshold, the graphene nanofillers are well apart, and the microcapacitor model is unlikely to be the main mechanism explaining the marked increase in the dielectric constant with graphene loading.^34^ The microscopic dipole model, as discussed earlier, is likely to be the contributing factor behind the dielectric constant enhancement observed in this work.

### Sensor Applications

#### Mechanism of Strain/Pressure Sensing

The piezocapacitive sensors proposed in this work utilize graphene–PVAc nanofibrous membranes as the dielectric layer. For a nanofibrous membrane-based capacitor with air voids, the initial effective dielectric constant is a combination of the dielectric constants of air and the nanofibrous material. Upon application of an external pressure, the gap between the electrodes reduces, thereby squeezing the sandwiched nanofibrous membrane and replacing the low permittivity air voids with high permittivity of the nanofibrous material in the process. This pressure-induced densification process increases the effective dielectric constant of the capacitor. The phenomenon of pressure-induced dielectric constant enhancement coupled with the usual thickness change significantly enhances the sensitivity of the sensor. Figure 3e schematically represents the mechanism of pressure-induced capacitance change utilized for the sensors reported in this work.

In the past, Kim et al. and Sharma et al. independently proposed analytical models to explain the dependence of capacitance change on applied external pressure.^24,33^ The relation between the external pressure (*p*) and capacitance change (Δ*C*) is given by the following expression
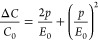
where *C*_0_ is the initial capacitance of the sensor and *E*_0_ is the Young’s modulus of the dielectric layer.^24^ For low pressure regimes, the applied pressure being less than the Young’s modulus (*p* ≪ *E*_0_), the quadratic term can be neglected, and the normalized capacitance change is expected to demonstrate a linear behavior
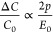
In contrast, for traditional MEMS capacitive sensors utilizing air gaps or bulk dielectric materials, the capacitance change is chiefly due to the change in thickness, and for the low pressure regime, the normalized capacitance change is
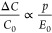


Kim et al. argued that, at low pressure regimes, the normalized capacitance change for nanofibrous piezocapacitive sensors is double that of their bulk dielectric material counterpart (without air voids).^24^ From the above discussion, it is evident that nanofibrous membranes with air voids are more likely to be suitable for the development of wearable piezocapacitive sensors.

**Figure 4:**
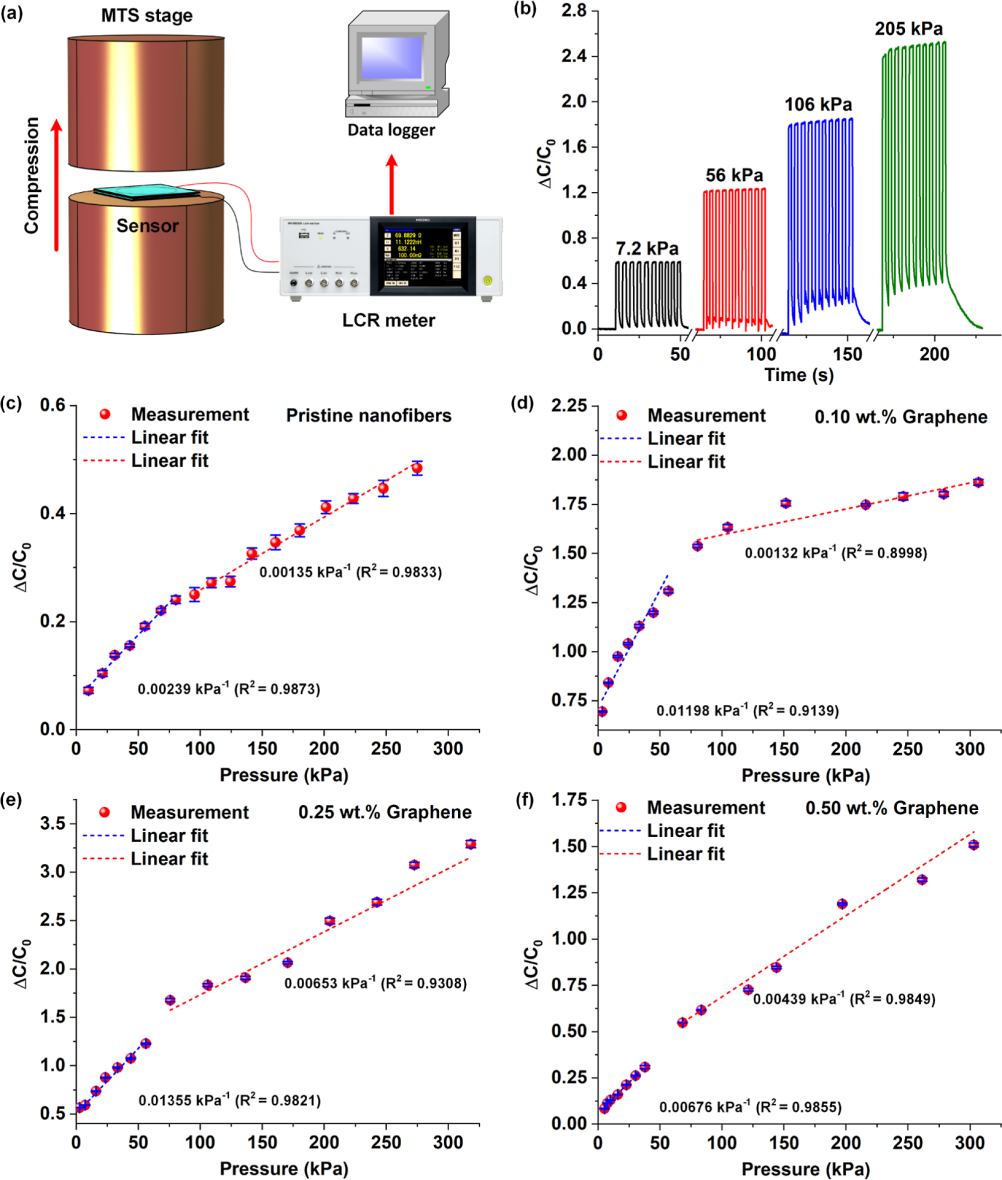
Electromechanical characterization of the nanofiber-based capacitive sensors: (a) schematic representation of the experimental setup for characterizing the pressure sensitivity of the capacitive sensors; (b) plot showing the normalized capacitance change responses of the 0.25 wt % graphene–PVAc pressure sensor for 10 cycles of four different uniaxial pressures (7.2, 56, 106, and 205 kPa); (c–f) individual plots showing the pressure-normalized capacitance change response of the pristine PVAc nanofiber and graphene-loaded (0.10, 0.25, and 0.50 wt %) PVAc nanofibers.

To assess the pressure sensing performance of the graphene–PVAc flexible pressure sensor, a series of tests involving tactile pressure sensing were performed. Figure 4a schematically represents the experimental setup employed for the electromechanical characterization experiment (more details are furnished in the Experimental Section). The bottom piston of the MTS 810 uniaxial testing setup was programmed to move at various fixed distances in a square wave fashion (at a frequency of ∼0.2 Hz) to apply cyclic pressure in the range ∼2–320 kPa. A series of square wave pressure loading-unloading tests comprising 10 cycles at 0.2 Hz were conducted on each of the individual sensors to determine their sensitivities. The plot in Figure 4b shows the normalized capacitance change response acquired with the 0.25 wt % graphene-loaded PVAc nanofiber-based sensor for four cyclic uniaxial pressure loadings of 7.2, 56, 106, and 205 kPa. The response and recovery times for the sensor were determined by employing loading pressure stimuli of 2.7 kPa. The response and recovery times were found to be ∼0.40 and ∼0.46 s respectively (Figure S1). The mismatch between the response and recovery times can be attributed to the hysteresis of the graphene–PVAc nanofibrous membrane. To further understand the effect of graphene nanofiller addition, the pressure sensitivity of the pristine PVAc nanofiber-based sensor was compared with that of the graphene-dispersed nanofiber sensors. The normalized capacitance change versus the applied uniaxial pressure was plotted, as shown in Figure 4c, and subsequent linear regression treatment revealed two separate linear regimes with pressure sensitivity figures of 0.00239 kPa^–1^ (in the pressure range 9.85–80.30 kPa) and 0.00135 kPa^–1^ (in the pressure range 95.45–275 kPa) with *R*-square values of 0.9873 and 0.9833, respectively. Similar uniaxial pressure sensitivity assessment tests were conducted on the graphene-dispersed PVAc nanofiber-based sensors, as shown by the plots in Figure 4d–f. Similar to the case of pristine PVAc nanofiber-based sensors, all the graphene-dispersed samples demonstrated two linear regimes. Linear regression treatment was conducted on the plots, and their pressure sensitivities (with R-squared values) are shown in the plots (Figure 4d–f). As discussed previously, sensors with nanofibrous membranes demonstrate a linear relation between the normalized capacitance change and applied pressure in lower pressure regimes. The experimental observations agree with our previously discussed analytical model. In general, the pressure sensitivities of the nanofibrous membranes increased with increasing graphene weight percentage. The reason behind the marked enhancement can be attributed to the enhancement of the dielectric constant with increasing filler concentration, as demonstrated previously in Figure 3c. The sensor utilizing 0.25 wt % graphene filler dispersion demonstrates the highest-pressure sensitivities for both the low- and high-pressure regimes. With higher graphene loading (0.50 wt %), a marked decrease in the pressure sensitivity (especially for low pressure regime) was observed, which can be attributed to the formation of graphene agglomerates and the possible increase of compression modulus with higher graphene loading, thus rendering the nanofibrous membranes stiffer and subsequently reducing the pressure sensitivity.^33^ The dynamic range, sensitivity, response/recovery times, and degradability of the sensor reported in this work are compared with recent similar piezocapacitive sensors in the literature and are presented in Table 1. Though some of the sensors reported in literature have very high sensitivity figures in ultra-low-pressure regimes, for the sake of a fair comparison, a pressure range of ∼1–400 kPa (relevant for the present work) was considered for contrasting them with the sensor reported in this work.

**Table 1:** Table Comparing the Sensitivity, Dynamic Range, Response Time, and Degradability of the Sensor Reported in the Present Work With Similar Sensors in the Literature

material	dynamic range (kPa)	sensitivity (kPa^–1^) & range (kPa)	response & recovery	degradability
PVDF-CNT nanofibers^36^	0–15	0.63 (1.2–15.0 kPa)	0.021 s	no
			0.029 s	
polyimide nanofibers^28^	0–1388	0.029 (0.56–13.89 kPa)	0.157 s	no
		0.000549 (13.89–1388.89 kPa)	0.175 s	
TPU nanofibers^29^	0–30	0.071 (4–30 kPa)	0.026 s	no
TPU nanofibers^48^	0–80	2.32 (1–5 kPa)	0.273 s	no
		0.65 (>5 kPa)	0.273 s	
PVDF-TrFE/MXene nanofibers^33^	0–400	0.01 (10–150 kPa)	0.15 s	no
		0.006 (150–400 kPa)	0.15 s	
TPU nanofibers^49^	0–40	0.085 (2–10 kPa)	0.065 s	no
		0.017 (10–40 kPa)	0.078 s	
TPU nanofibers^50^	0–70	0.029 (0–10 kPa)	0.26 s	no
		0.015 (10–70 kPa)		
Graphene–PVAc nanofibers^this work^	0–320	0.014 (2.73–56.06 kPa)	0.40 s	yes
		0.006 (75.76–318.18 kPa)	0.46 s	

**Figure 5:**
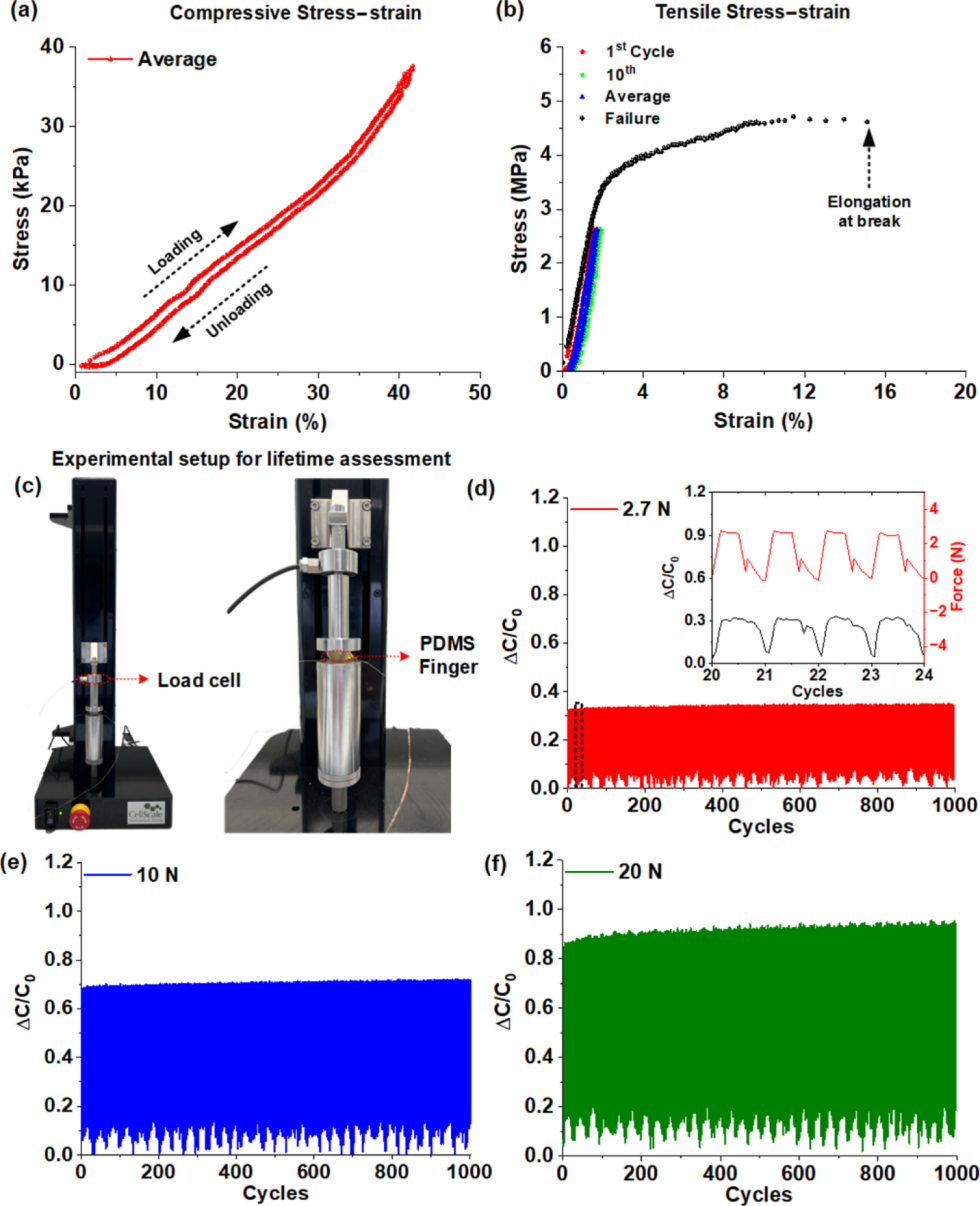
Mechanical characterization and accelerated lifetime assessment: (a) plot showing the average compressive stress–strain characteristics of the 0.25 wt % Graphene–PVAc nanofiber-based sensor; (b) plot comparing the tensile stress–strain curve of 1^st^ and 10th cycles with the average stress–strain characteristics (averaged over 10 cycles). The plot also shows the ultimate tensile strength of the nanofiber membrane and its elongation at break. (c) Experimental setup for accelerated lifetime assessments; (d) Relative capacitance change response of the sensor to 1000 cycles of 2.7 N tactile force loading with an artificial PDMS finger. Magnified view of the plot comparing the sensor response with the force input curve in the period 20–24 cycles; (e,f) Relative capacitance change response of the sensor to 1000 cycles (each) of tactile force loadings of 10 and 20 N, respectively.

Owing to its maximum pressure sensitivity, the 0.25 wt % graphene–PVAc nanofiber-based sensor was chosen for further mechanical and lifetime assessment analyses. To assess the compressibility and hysteresis behavior, the sensor assembly was subjected to a series of 10 cyclic compressive loading and unloading. Further details of the test setup are furnished in the Supporting Information section (Figure S2a). The plot in Figure 5a shows the stress–strain characteristics of the sensor assembly averaged over 10 cycles of periodic loading and unloading. The hysteresis (%) was determined by using the following expression^51^
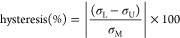
where  and  represent loading and unloading stresses, respectively, at a particular strain, and  represents the maximum stress. The hysteresis values were determined as ∼ 4.31059, 4.93055, 3.61668, 3.61668, and 2.84959% for ∼5, 10, 20, 30, and 40% compressive strains, respectively, at a maximum pressure of 37.17 kPa. The relatively low hysteresis demonstrated by the sensor for repetitive compressive loading underscores its resilient nature, excellent recoverability, and suitability for pressure sensing.

Furthermore, the tensile modulus and ultimate tensile strength of the 0.25 wt % graphene-loaded PVAc nanofiber membrane were assessed by subjecting it to a series of cyclic tensile loading and unloading. Further details of the test setup are furnished in the Supporting Information section (Figure S2b). The plot in Figure 5b compares the tensile stress–strain curve of the 1^st^ and 10th cycles with the average stress–strain characteristics (averaged over 10 cycles). The average tensile modulus of the nanofiber membrane was determined as 212.69 MPa with an ultimate tensile strength of 4.61 MPa at 15.10% strain (elongation at break).

To demonstrate the robustness and reliability of the sensor for long term applications, accelerated lifetime assessment experiments involving a combined total of 3000 cycles of tactile force loading at three different force levels (2.7, 10, and 20 N) were conducted. The photograph in Figure 5c shows the experimental setup employing the CellScale Univert mechanical testing stage (fitted with a 100 N load cell) employed for the lifetime assessments. To emulate real-life applications involving a human finger–sensor interface, an artificial PDMS finger was attached to the top movable piston, and the 0.25 wt % graphene-loaded PVAc nanofiber-based sensor was secured on the bottom fixed stage. A 0.75 N preload was applied to the sensor to prevent measurement artifacts during lifetime assessment. The movable piston was programmed to apply a series of cyclic loads (entailing square wave pulses with a ramp up/down time of 0.25 s and a cycle period of 1.5 s). The plots in Figure 5d–f show the relative capacitance change responses of the sensor to 2.7, 10, and 20 N loadings of 1000 cycles each. The inset plot in Figure 5d shows the magnified view of the sensor response (black) between cycle numbers 20 and 24 and compares it with the force input curve (red). The peculiarity in the load curve while ramping down and subsequent relaxation can be attributed to the viscoelastic property of the PDMS finger model that was employed for transferring the desired load to the piezocapacitive sensor. As observed from the plots, the sensor maintains its relative capacitance change characteristics without any significant degradation, thus underscoring its robustness and long-term reliability. Due to their superior performance and robustness, the 0.25 wt % graphene-dispersed PVAc nanofibers were chosen for further experiments involving human physiological monitoring applications due to their excellent pressure sensing performance.

#### Applications of the Graphene–PVAc Nanofiber Sensor

**Figure 6:**
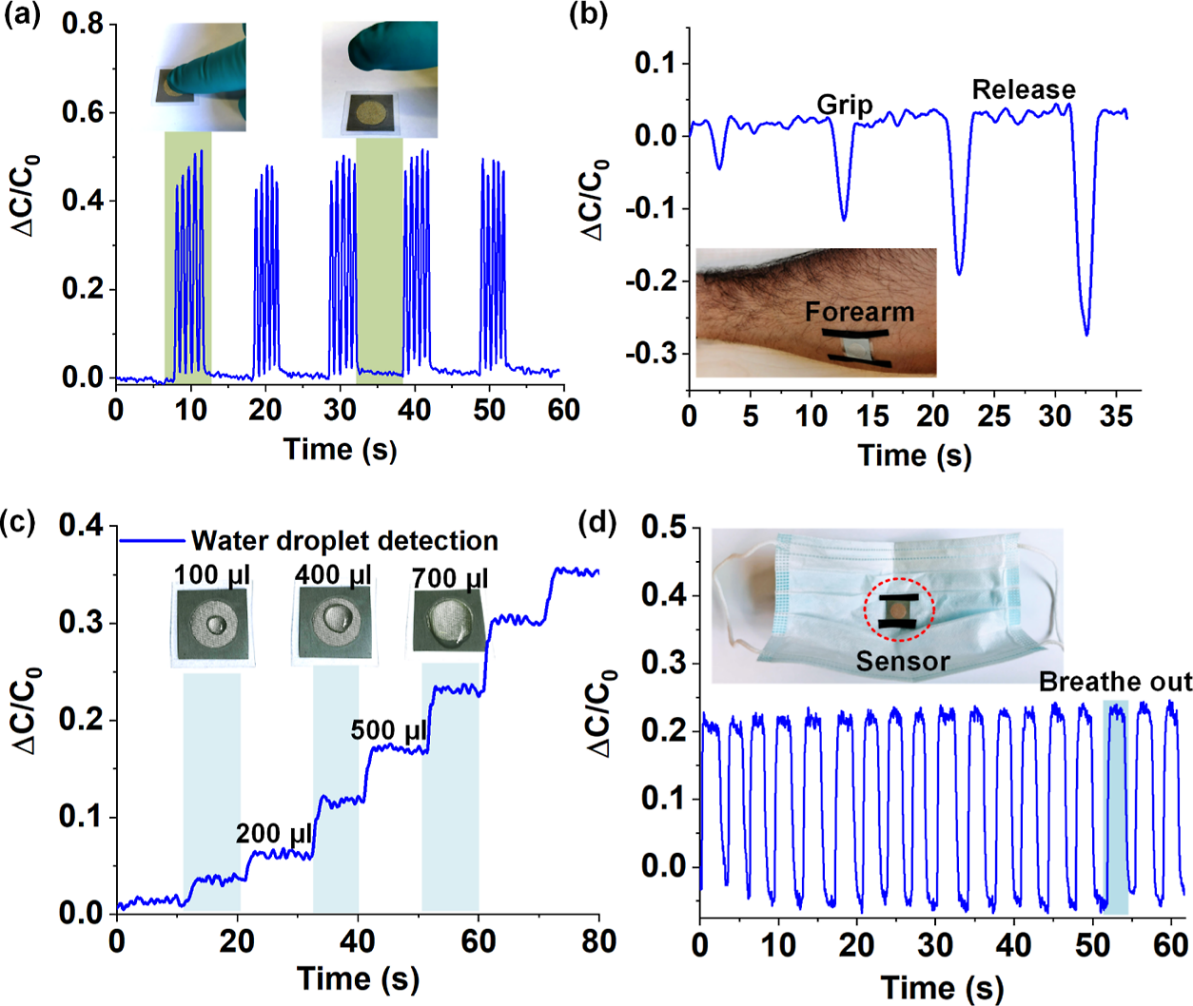
Application of the 0.25 wt % graphene–PVAc nanofiber capacitive sensors for human physiological monitoring: (a) plot demonstrating the capability of the sensor in identifying finger tapping, which is relevant for Parkinson’s disease patients having abnormal finger tremor; (b) plot showing the response of the sensor to forearm muscle activation; (c) response of the capacitive sensor to water droplet loading in steps of 100 μL; (d) response plot of the sensor (attached to the inner lining of a surgical mask) to normal breathing.

To demonstrate the applicability of our piezocapacitive sensors for physiological function monitoring applications, the 0.25 wt % graphene-dispersed nanofiber-based sensors were chosen owing to their superior pressure sensing performance, as demonstrated previously. The plot in Figure 6a shows the capability of the graphene–PVAc nanofiber sensor in the detecting index finger tapping movements (emulating low-frequency finger tremor). In another experiment, the sensor was secured on the ventral arm muscle region of the right arm, as shown in the inset of Figure 6b. The muscle was activated by gripping a stress ball with the right fist in periodic fashion at different grip intensities. As shown by the plot in Figure 6b, the griping action produced a sharp peak. This exercise was repeated four times at increasing grip intensities, and the amplitude of the sensor increased with the grip intensity. To demonstrate the capability of the sensor in detecting the minutest pressure change, a water droplet measuring as little as 100 μL was loaded on the sensor, and the normalized pressure change response was measured. The droplets were increased in steps, and the sensor response was plotted as shown in Figure 6c. The applicability of the sensor in respiratory rate monitoring was demonstrated by attaching the sensor to the inner lining of a disposable surgical mask and conducting subsequent tests. A healthy test subject (age 30 years) was made to wear the mask and breathe deeply, and the response of the sensor was plotted, as shown in Figure 6d. The breathing rate (BRPM) was found to be ∼18 BRPM, and the result was matched with a commercial fitness activity monitoring device (Garmin Fenix 6).

#### Piezocapacitive Sensorized Shoe Insole

**Figure 7:**
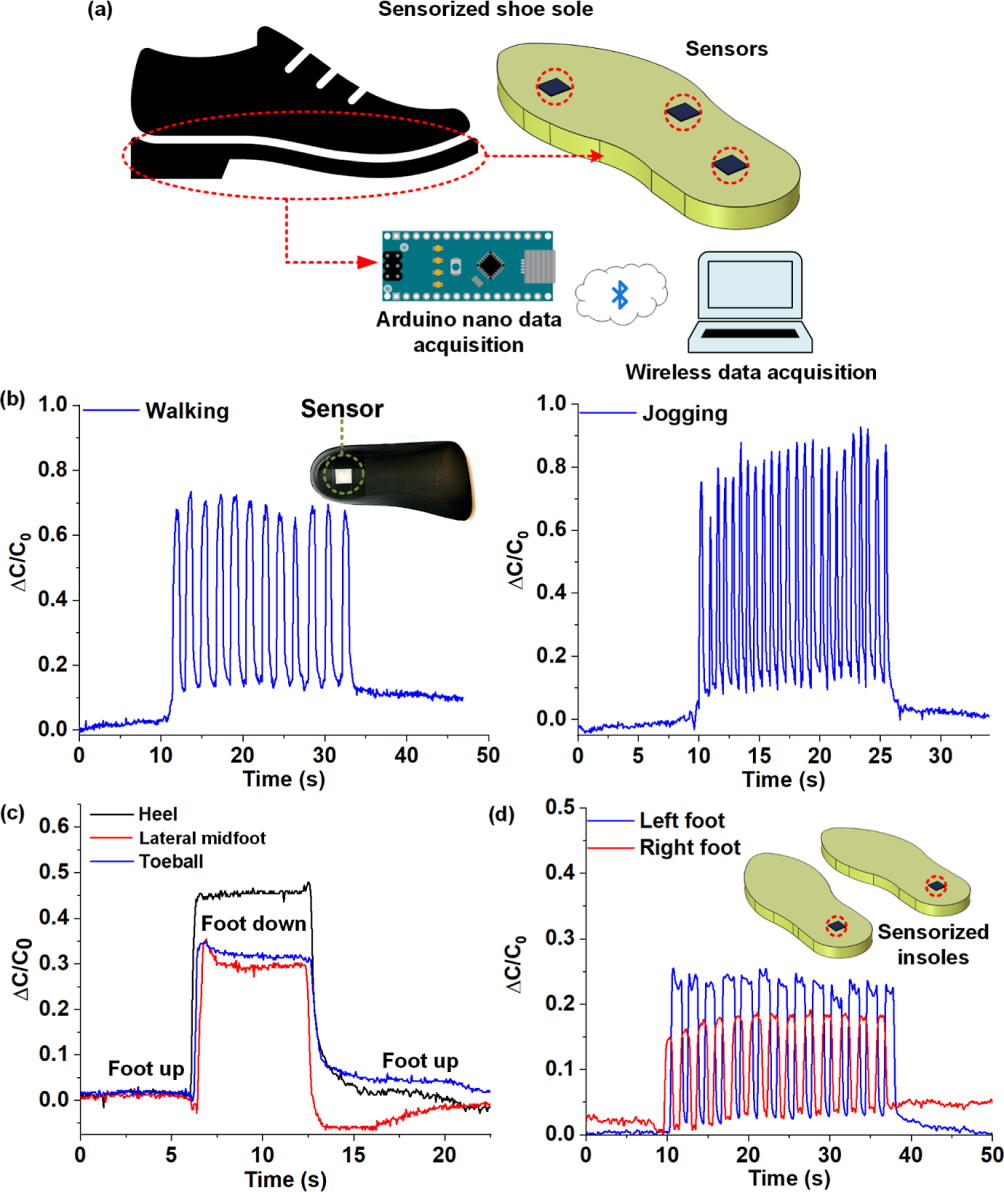
Sensorized shoe sole for gait monitoring: (a) schematic representation of the sensorized shoe sole for gait monitoring; (b) plots comparing the responses of the sensor from the heel region while walking (left) and jogging (right); (c) plot showing qualitative pressure beneath the right foot using the sensorized shoe sole; (d) plots showing the gait pattern of a healthy subject while walking with a pair of sensorized soft insoles (with sensors placed in the heel region).

In the past, Cavanagh et al. have reported standing plantar pressure distribution under human feet for a heterogeneous sample of feet (*N* = 107).^52^ Peak plantar pressures were reported to lie in the range 53–139 kPa. In our previous research, we have demonstrated utilizing graphene–PDMS squeezable sensors for developing smart sensorized shoe soles for gait monitoring applications.^53^ As seen previously, the sensors presented in this work have been calibrated for pressure sensitivities in the range of ∼2–320 kPa that is the regime of interest for plantar pressure sensing. Here, we have utilized the graphene–PVAc nanofiber sensors for demonstrating IoT-enabled gait monitoring. As discussed previously, capacitive sensors are more suitable due to their low hysteresis, fast response, and low power budget. Three identical sensors were secured on the heel, lateral midfoot, and toe ball regions of a soft shoe insole and placed inside a sport sneaker. For data acquisition, embedded systems software utilizing the Arduino Nano platform was employed. The details of the connections are furnished in the experimental section. For wireless data acquisition, an Arduino HC05 Bluetooth module utilizing the serial communication protocol was used. The IoT enabled system utilizing the Arduino nanoplatform is schematically represented in Figure 7a. The plots in Figure 7b show the sensor response from the heel region while a subject walked and jogged (the LCR meter was used for data acquisition in this case). The photograph in the figure inset shows the placement of the actual sensor in the heel region of a shoe insole. A clear distinction could be made between the walking and jogging gaits based on the signal frequency that represents heel strike. The sensorized insole with the three sensors secured on the heel, lateral midfoot, and toe ball regions was utilized for demonstrating the ability of the system in qualitative pressure distribution monitoring. A healthy test subject (age 30) weighing 60 Kg was asked to wear a shoe with the sensorized sole placed inside. The data was logged while the right foot was lifted for six s followed by the right foot down (standing position) for ∼6 s before finally lifting off the right foot. The plot in Figure 7c shows the sensor responses acquired from the heel, lateral midfoot, and toe ball region while the footstep experiment was conducted. At regular standing posture (foot down), the sensor in the heel region showed maximum peak intensity, followed by the sensors at the toe ball and lateral midfoot. Cavanagh et al. reported average peak pressures of 139 kPa under the heel, significantly higher than the average peak pressure under the forefoot, which was found to be 53 kPa. Finally, two graphene–PVAc sensors were secured at the heel regions of a pair of shoe insoles and placed inside a pair of casual shoes. The plot in Figure 7d shows the responses acquired from the sensors while walking in a straight line. The plot clearly shows the phase lag between the sensor signals from the left and right heels while walking. Though the data from only the heel region might seem rudimentary at first glance, important secondary data like ground reaction force and cadence can be derived further if parameters like step length and weight are known. For instance, in this experiment, the cadence is determined to be 52 steps per minute. The subject has a measured step length of 60.96 cm (2 feet) and a stride length of 121.92 cm (4 feet). The walking speed of the test subject is calculated as 31.97 m min^–1^ (determined by multiplying the step length by cadence). The sensorized shoe insole demonstrated in this work can potentially be used by physicians to determine problems with gait and determine if further diagnostic tests are warranted to find the underlying condition.

#### Sitting Posture-Monitoring Ergonomic Smart Chair

**Figure 8:**
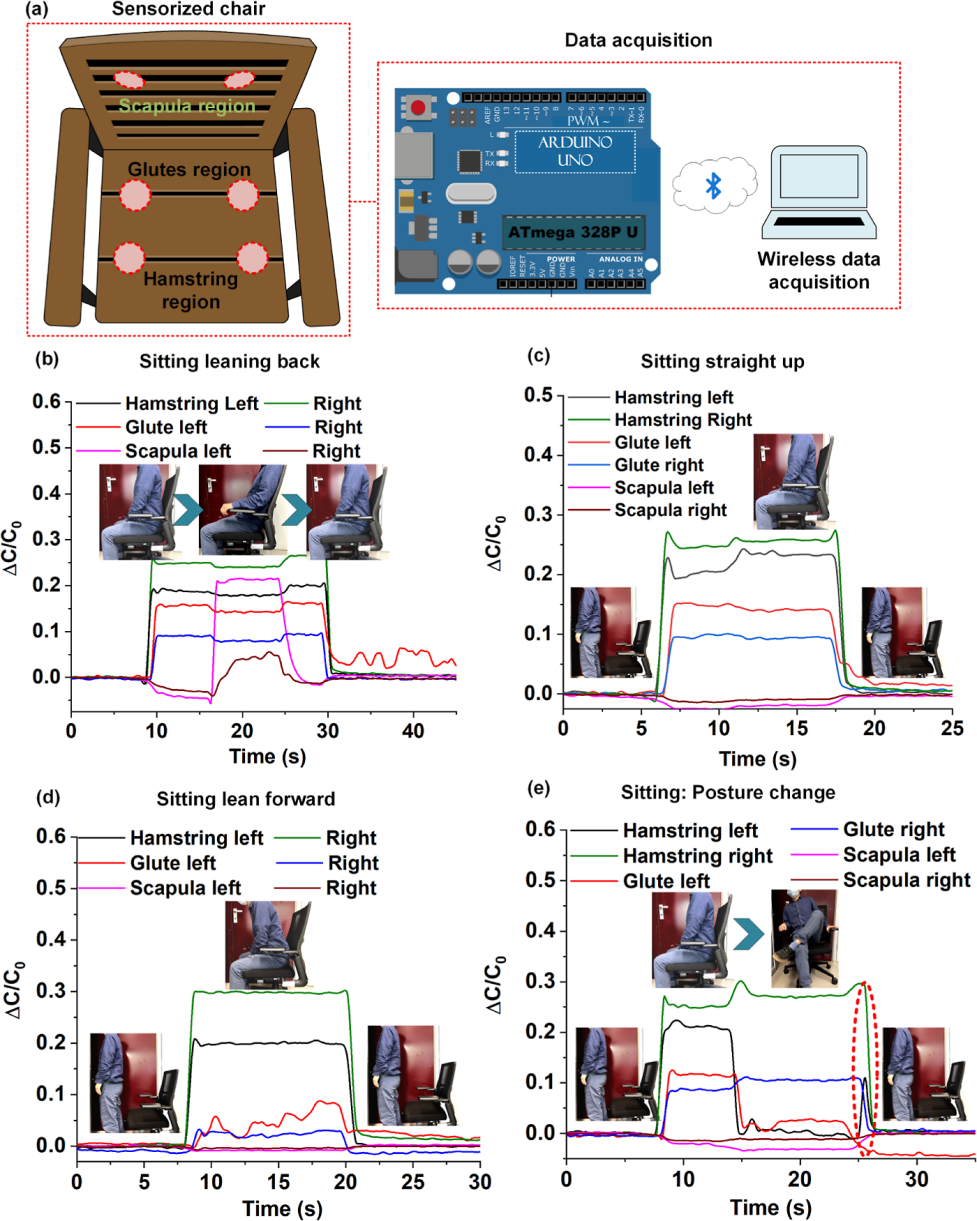
Posture-sensing smart chair: (a) schematic representation of the smart chair and Arduino-based data acquisition setup with the position of the capacitive sensors for qualitative pressure monitoring; (b) plot showing qualitative pressure distribution while a person sits upright versus sitting with back leaning against the support; (c) plots showing the qualitative pressure profile while a subject sits upright; (d) plot showing sensor responses while a subject sits leaning forward; (e) plots showing the sensor responses while a subject changes posture while sitting.

Here, we demonstrate the applicability of the graphene–PVAc sensors for ergonomic smart chair systems. The system entailed six pressure sensors placed on bilateral hamstrings, gluteal, and scapular regions on an office chair, as shown schematically in Figure 8a. The sensors were coupled to an embedded system software implemented on an Arduino Uno board for IoT readiness. A healthy subject (age 28, weight 57 kg) was asked to sit comfortably on the chair and adapt various postures while the normalized capacitance change data was acquired using the embedded systems software. The plot in Figure 8b shows the sensor responses acquired while the user sat up right for approximately 5 s followed by leaning back on the back support for 10 s, and sitting upright (with the upper back off the backrest) for 5 s. The bar plots below the line plot show qualitative pressure distribution over the period of 16–26 s. The subject was made to repeat a modified exercise wherein the subject stood for 5 s followed by sitting down upright (without upper back on the back support) for approximately 12 s before finally standing up. The plot in Figure 8c shows the sensor responses acquired while the sitting exercise was performed. As expected, no significant change in signal was observed from the sensors placed in the scapulary region of the chair backrest. The plot in Figure 8d shows the sensor responses while the test subject sat down leaning forward. The pressure distribution was observed to be maximum under the bilateral hamstrings, whereas the pressure distribution under the bilateral gluteal regions was significantly reduced in comparison to previous two cases (Figure 8b,c). As observed previously, no significant changes were observed from the bilateral scapulary sensors. Finally, the test subject was asked to perform a posture-changing maneuver while sitting in the chair. The subject sat upright for 5 s (upper back off the backrest), followed by a lifting of the right hamstring to sit in a folded leg posture (shown in figure inset) for approximately 10 s before finally standing up. The plot in Figure 8e shows the responses acquired from the sensors during the sitting and posture change activities. The sensor system was sensitive and fast enough to show the minute change in posture (left hamstring down to normal upright sitting position) before standing up, as shown by the dotted ellipse (centered around 26 s) in Figure 8e. The experiments involving the posture-sensing smart chair further underscore the applicability of our piezo capacitive sensors for nonintrusive human physiological signal sensing applications.

#### Degradability

**Figure 9:**
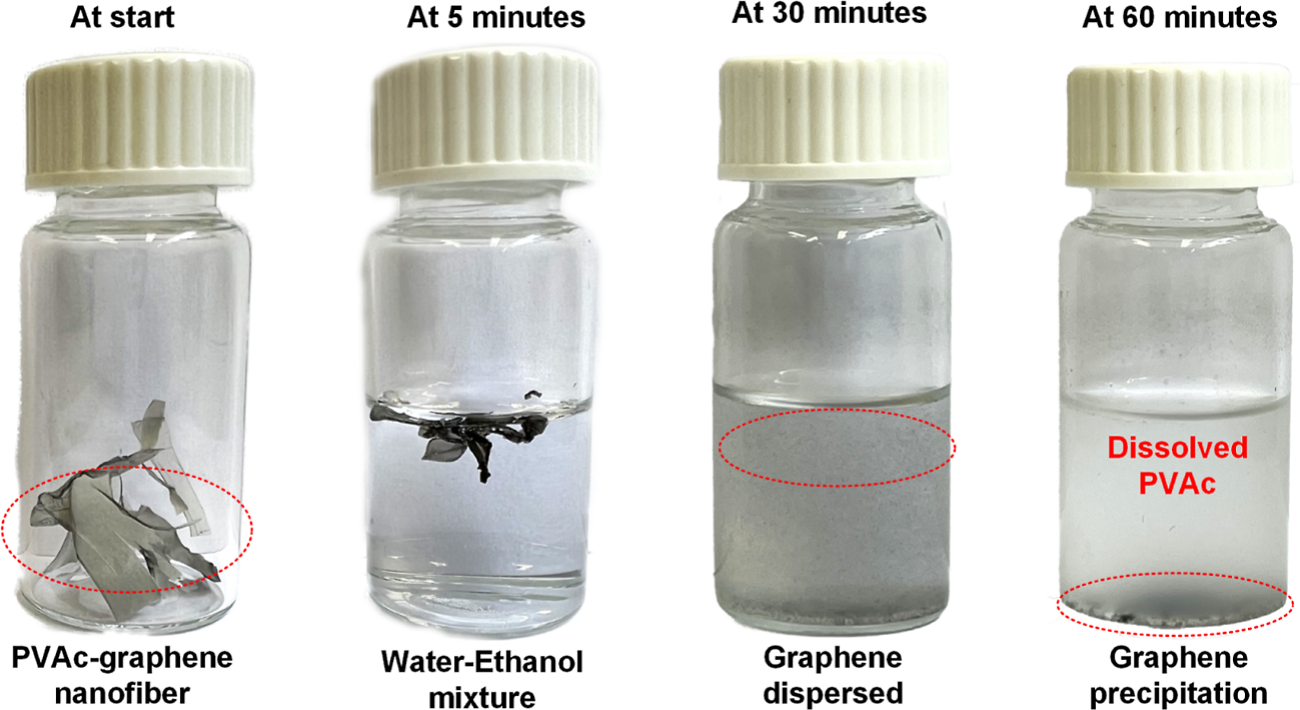
Photographs showing the degradation of the 0.25 wt % graphene–PVAc nanofibrous membranes at different points in time.

With the growing burden of electronics waste lately and their detrimental impact on the environment and public health, there is a surge in demand for transient electronics.^54^ To show the easy degradability and recyclability of the sensing elements, 0.25 wt % graphene-loaded PVAc nanofibers were placed in a glass vial with a 10 mL solution of deionized water and ethanol (ratio 1:1). The solution was stirred using a hot plate magnetic stirrer at 60 °C to accelerate the decomposition process. The large specific surface area of the nanofibrous membrane provides more area for the water–ethanol solution to penetrate, thereby facilitating the decomposition process. At the end of 30 min, clear graphene dispersion in the solution was observed. The solution was removed from the stirrer and allowed to cool to room temperature. By the end of 60 min, graphene precipitation was observed, and the solution turned whitish, indicating a homogeneous solution of PVAc in the water–ethanol mixture. The photographs in Figure 9 show the nanofibrous membranes in the water–ethanol solution at different points in time. With further treatment, graphene nanoflakes could be separated from the PVAc solution, thus demonstrating the seamless integration of the proposed sensing element for the circular electronics theme.

Though the PVAc-graphene nanofiber membrane is degradable, the lack of suitable degradable packaging restricts the applicability of the reported sensor for applications demanding true, fully degradable sensors. To make the sensor fully degradable yet robust and reliable, water-soluble packaging materials like polyvinyl alcohol (PVA), polyethylene glycol (PEG), and water-soluble hydrogels can be considered in the future. Furthermore, conductive polymer-based flexible electrodes^33^ can be used in conjunction with flexible and biodegradable packaging to circumvent the recyclability issue often associated with deposited metal-based thin film electrodes.

## Conclusions

In conclusion, this work established a facile method of utilizing graphene-dispersed PVAc nanofibrous membranes for IoT-enabled wearable piezocapacitive sensing applications. A series of electrical and material characterization tests involving dielectric response assessment, SEM and TEM observation, and Raman spectral analyses were conducted on the nanofibers to understand the effect of graphene dispersion on nanofiber morphology, and electrical properties. Microcapacitor network and microdipole formation mechanisms were invoked to explain the observed nanofiller-induced dielectric constant enhancement. A series of dynamic uniaxial pressure sensing performance tests were conducted on the pristine and graphene-dispersed PVAc nanofibrous membrane-based piezocapacitive sensors to observe and understand the effect of graphene nanofiller addition on pressure sensing performance. The nanofibrous membrane with 0.25 wt % graphene loading showed superior pressure sensing performance with two distinct linear regimes and pressure sensitivity figures of 0.01355 kPa^−1^ (∼2.73–56.06 kPa) and 0.00653 kPa^–1^ (∼75.76–318.18 kPa). A series of accelerated lifetime assessment tests involving at least 3000 cycles of tactile force loading at three different forces were conducted to demonstrate the robustness and reliability of the sensor. Finally, the 0.25 wt % graphene-dispersed PVAc nanofibrous membrane-based sensors were chosen for demonstration experiments. A series of human physiological parameter monitoring experiments comprising muscle movement detection, respiratory rate detection, and gait monitoring were conducted. To further emphasize the readiness of the sensors for IoT applications, a wireless smart gait monitoring shoe insole and a posture sensing smart chair, coupled with a custom embedded program implemented on Arduino Uno and nanoplatforms were demonstrated. Finally, the suitability of the proposed sensing element for the circular electronics initiative theme has been demonstrated through the simple decomposition of the sensor in a water–ethanol solution.

## Experimental Section

### Electrospinning of PVAc and Graphene–PVAc Nanofibers

For electrospinning of pristine PVAc and graphene–PVAc nanofibers, PVAc beads (*M*_W_ 500,000 g mol^–1^), *N*,*N*-dimethylformamide (DMF), and electrochemically exfoliated graphene ink (1 mg mL^–1^ in DMF) purchased from Sigma-Aldrich, the Netherlands, were used. For the pristine PVAc nanofibers, 1.5 g of PVAc was dissolved in 10 mL of DMF and stirred at room temperature using a magnetic stirrer to achieve 15% (w/v) solution. For the graphene–PVAc nanofibers, the commercially acquired graphene dispersion was added to the PVAc polymer solution and subsequently sonicated employing an ultrasonic bath sonicator to achieve 0.1, 0.25, and 0.50% weight of graphene nanofillers in PVAc, while keeping the overall concentration of the solution fixed at 15% (w/v). An electrospinning setup (NanoSpinner NE300) from Inovenso electrospinner fitted with a stainless-steel rotating mandrel collector was used for electrospinning the nanofibers. Inovenso IPS-14RS syringe pump fitted with a 10 mL syringe was used to feed the polymer solution through a needle (20 G) at a constant flow rate of 1 mL h^–1^. The electrospinning was conducted for the pristine PVAc solution and different concentrations of the graphene-loaded solutions by applying a potential difference of 15 kV between the tip of the needle and the rotating mandrel collector (rotating at 2200 rpm) separated by 15 cm. The electrospinning process was conducted for 3 h to obtain a film of uniform nanofibers deposited on the aluminum foil substrate.

### Characterization of the Nanofibers

For understanding the diameter distribution and morphological characteristics of the various electrospun nanofibers, FEI Nova NanoSEM 230 field-emission scanning electron microscopy (FE-SEM) was employed. Samples having dimensions of 1 cm × 1 cm were sputtered with gold and placed on appropriate SEM stubs for subsequent imaging. For conducting the imaging experiments, an acceleration voltage of 7 kV and a spot size of 3.0 were employed.

### Compositional Analyses

To confirm the dispersion of the graphene nanoflake fillers within the PVAc nanofibers, Raman spectroscopic analyses and TEM imaging were conducted. Raman spectra analysis was carried out on a PerkinElmer Raman station by using 633 nm laser excitation. Raman analyses were conducted on both the pristine and graphene-dispersed PVAc nanofibers. For the TEM measurements, the specimens were prepared by directly transferring the nanofibers from the aluminum foil to copper grids. Bright-field imaging was performed with a JEOL 2010 at an accelerating voltage of 200 kV.

### Electrical and Pressure Sensing Characterization

The fabrication flow for developing the pristine PVAc and graphene–PVAc nanofiber pressure sensors is schematically represented in Figure 1a. The nanofiber membrane was sandwiched between two layers of circularly shaped (radius ∼6.5 mm) silver-plated nylon fabric and electrically bonded to single-strand copper wire. Finally, complete encapsulation was achieved by employing two optically clear plate seals (acquired from Thermo Fisher). For electrical characterization experiments involving capacitance, dielectric constant, and pressure sensing performance, a Hioki IM3536 LCR meter was employed. For characterizing the pressure sensitivity of the pristine PVAc and graphene–PVAc nanofiber sensors, an MTS 810 uniaxial testing setup fitted with an external load cell was employed. The sensors to be evaluated were affixed to the bottom movable piston of the experimental setup with double-sided tape. The setup was programmed to apply cyclic (square wave stimuli at a frequency of 0.2 Hz) pressure in the range ∼2–320 kPa. The data from the pressure sensing characterization experiments were acquired employing a Hioki IM3536 LCR meter with an AC bias of 1 V at 1 kHz.

### Human Motion Monitoring and Sensor Demonstration Experiments

For demonstrating the capability of the sensor in IoT-enabled human physiological monitoring applications, a custom-made capacitance meter utilizing the Arduino Uno and Arduino Nano platforms was developed. The embedded system setup utilizes an open-source Arduino capacitor library that enables the measurement of capacitance in the range 0.2 pF–100 μF. For measurement and sensing experiments, each individual sensor was connected to an analog pin and a digital pin of the Arduino Uno/Nano board. For data acquisition using the Arduino platform, an open-source plug-in code (Ardu Spreadsheet 1.0)^55^ was employed for exporting the data from the Arduino serial output to CSV format. For all the experiments involving human motion monitoring and the smart chair, unamplified signal outputs were presented in the article.

## Supporting Information Available

Damage of a PVAc nanofiber fragment caused by progressive irradiation thereby exposing the embedded graphene nanoflakes (MP4)
Response and recovery times of the 0.25 wt % graphene–PVAc nanofiber-based sensor, cyclic compressive stress–strain characteristics, and tensile stress–strain characteristics (PDF)
