# Supplementary material for: Fabric-like Electrospun PVAc–Graphene Nanofiber Webs as Wearable and Degradable Piezocapacitive Sensors
Source: ACS Appl Mater Interfaces. 2023 Apr 25;15(18):22351–66. doi: 10.1021/acsami.3c03113 (PMC10176318; doi:10.1021/acsami.3c03113)
Supplement: Supplementary file 2 — am3c03113_si_002.pdf [file am3c03113_si_002.pdf]

Supporting information

Fabric-like electrospun PVAc-graphene nanofiber webs as wearable and degradable piezocapacitive sensors

Debarun Sengupta[1], Liqiang Lu[1], Diego Ribas Gomes[1], Bayu Jayawardhana[2], Yutao Pei[1], and Ajay Giri Prakash Kottapalli[1] *

[1]Department of Advanced Production Engineering (APE), Engineering and Technology Institute Groningen (ENTEG), University of Groningen, Groningen 9747 AG, The Netherlands

[2]Department of Discrete Technology and Production Automation, Engineering and Technology Institute Groningen, Faculty of Science and Engineering, University of Groningen, Groningen 9747 AG, The Netherlands

Corresponding author: a.g.p.kottapalli@rug.nl (Ajay Giri Prakash Kottapalli)

The response and recovery times of the 0.25 wt% graphene-PVAc nanofiber based sensor were determined by employing 2.7 kPa pressure loading stimuli. From the experiment, the response and recovery times of the sensor were determined as ~ 0.40 and 0.46 s respectively. The slightly longer recovery time can be attributed to the hysteresis in the nanofibrous membrane. The plots below in Figure S1 show the response and recovery times of the sensor.

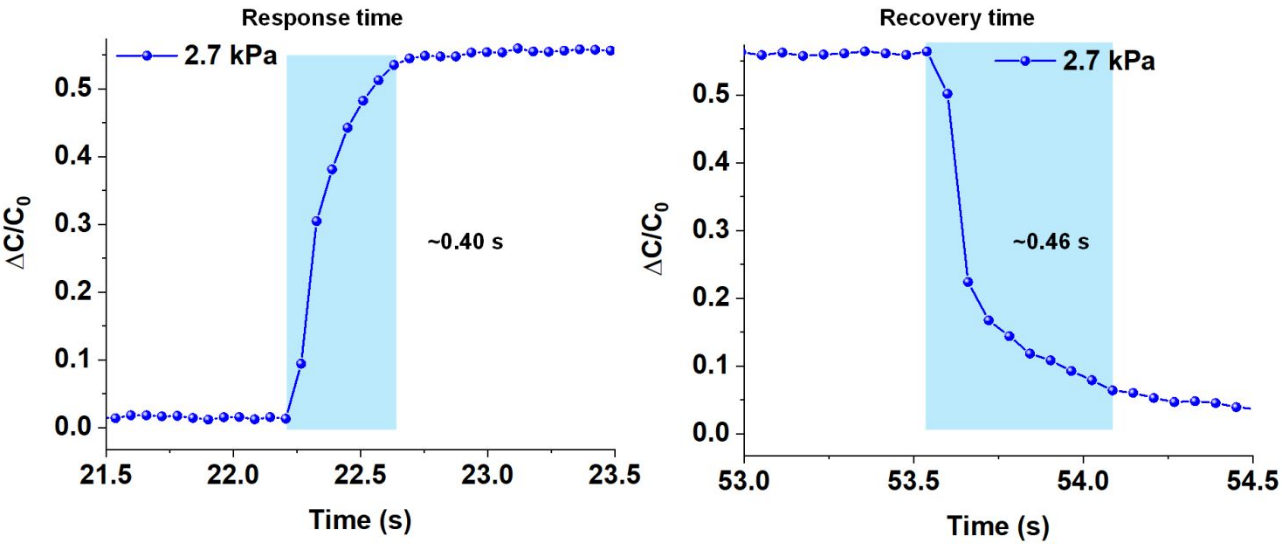

Figure S1: Plots showing the response and recovery times for the 0.25 wt% graphene-PVAc nanofiber based sensors.

To assess the compressibility and hysteresis behavior, the sensor assembly was subjected to a series of 10 cyclic compressive loading and unloading employing CellScale Univert mechanical testing stage (fitted with a 100 N load cell). The setup was programmed to apply the loading unloading cycles with a time period of 80 seconds (ramp up – 20 seconds, hold -20 seconds, ramp down -20 seconds, and rest – 20 seconds). The plot in Fig. S2a, shows the compressive stress-strain characteristics of the sensor for 1st, 2nd, 4th, 6th, 8th and 10th cycles.

Furthermore, the tensile modulus and ultimate tensile strength of the 0.25 wt. % graphene loaded PVAc nanofiber membrane were assessed employing the same experimental setup described previously. A 10 N load cell was used for the tests. The setup was programmed to apply a series of 10 tensile loading-unloading cycles with a time period of 20 seconds (ramp up – 5 seconds, hold -5 seconds, ramp down -5 seconds, and rest – 5 seconds). The plot in Fig. S2b, shows the tensile stress-strain characteristics of the sensor for 1st, 2nd, 4th, 6th, 8th and 10th cycles.

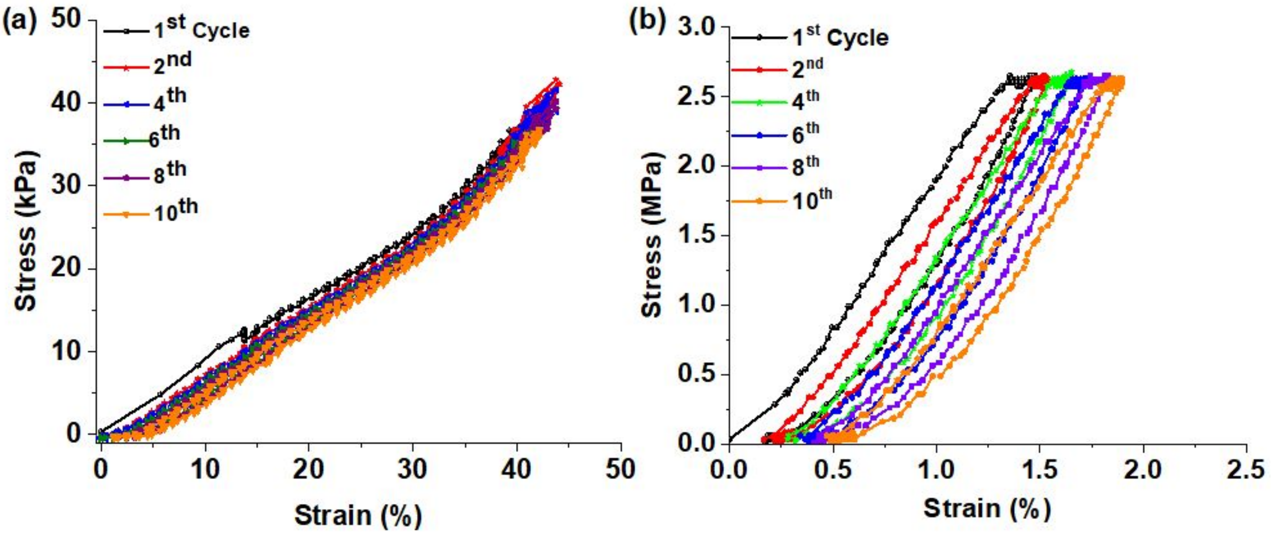

Figure S2: (a) Plot showing the compressive stress-strain characteristics of 1st, 2nd, 4th, 6th, 8th, and 10th cycles of the 0.25 wt.% graphene-PVAc nanofiber based sensor assembly; (b) Plot showing the tensile stress-strain characteristics of 1st, 2nd, 4th, 6th, 8th, and 10th cycles of the 0.25 wt.% graphene-PVAc nanofiber membrane without the encapsulation.
